# Olga von Leonowa (1851–?): eine Neurowissenschaftlerin verschwindet

**DOI:** 10.1007/s00115-020-01009-5

**Published:** 2020-10-06

**Authors:** Hans Förstl

**Affiliations:** Klinik und Poliklinik für Psychiatrie und Psychotherapie, Technische Universität München, Ismaningerstr. 22, 81675 München, Deutschland

Auf dem Gruppenfoto steht eine einzige Frau zwischen mehr als fünfzig Männern der Sektion für Psychiatrie und Neurologie, die 1894 an der 66. Versammlung deutscher Naturforscher und Ärzte in Wien teilnahmen: Olga Vassilievna von Leonowa (Abb. 1). Sigmund Freud scheint in der zweiten Reihe demonstrativ von ihr abzurücken [6]. Von Leonowa hatte die Vormittagssitzung des 25. September unter dem Vorsitz von Geheimrath Professor Friedrich Jolly aus Berlin mit ihrem Referat über „die Sinnesorgane und die Ganglien bei Anencephalie und Amyelie“ eröffnet [12]. Danach diskutierten ihr Züricher Lehrer Konstantin von Monakow, Auguste Forel, er fügte eine kleine historische Korrektur an, und Arnold Pick, der auf einen ähnlich gelagerten Fall verwies, den er 1877 auf der Münchener Naturforscherversammlung vorgestellt hatte. Gleich im Anschluss hielt Forel den Hauptvortrag zur Rolle des Alkohols bei Epilepsie und Perversionen aller Art, gefolgt von Aloys Alzheimers Vortrag über die progressive Paralyse. Von Leonowas Beitrag wurde als so wichtig erachtet, dass nicht nur das *Neurologische Centralblatt* [12], sondern die *Wiener Neue Freie Presse* gleich am nächsten Tag darüber berichtete [8]. „Von besonderem entwicklungsgeschichtlichen Interesse war ein Vortrag von Frl. Dr. v. Leonowa, einer russischen Ärztin, … über die Entwicklung des Nervensystems bei menschlichen Früchten ohne Gehirn und Rückenmark. Ihre anatomischen Untersuchungen haben ergeben, dass bei solchen Früchten zwar die Entwicklung der motorischen Nerven unterbleibt, diese also vom Rückenmark ausgeht, dass aber die Empfindungsnerven und die sympathischen sich, trotzdem kein Rückenmark da ist, aus seitlichen Ganglien-Anlagen entwickeln, ja, es kam bis zur Bildung einer Netzhaut, freilich ohne Nervenfasern. …“ [8].

**Figure 1:**
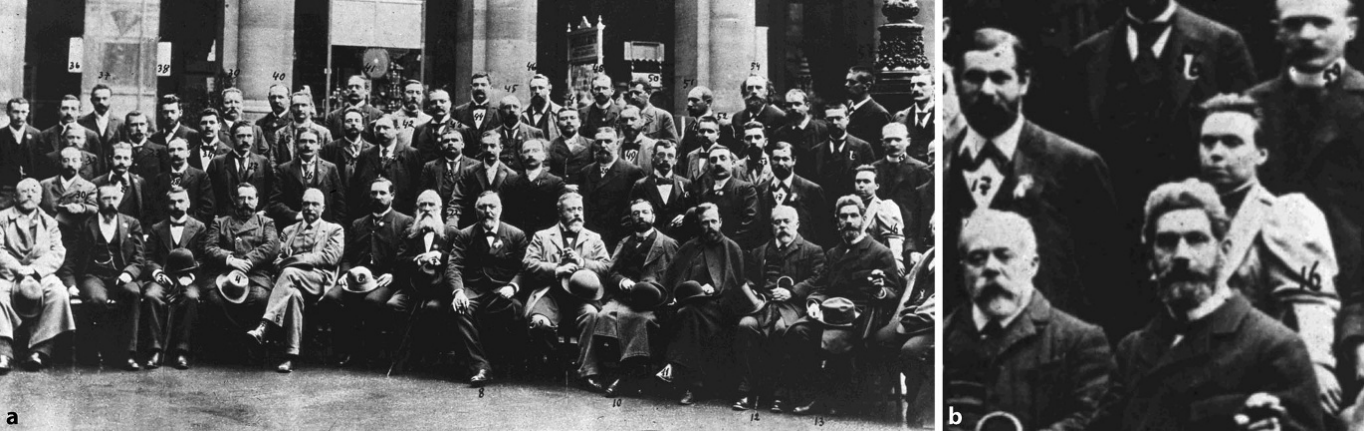

## Entwicklungsgeschichte und Arbeit

Olga von Leonowa hatte nach eigener Angabe das Medizinstudium mit 28 Jahren an der Kaiserlichen Universität in Moskau begonnen und in Sankt Petersburg, Wien und Leipzig fortgesetzt [5, S. 21]. Russischen und US-amerikanischen Studentinnen war das Medizinstudium bereits erlaubt und sie wurden ermutigt ihren Horizont in Westeuropa zu erweitern und einige, die es sich leisten konnten, machten davon regen Gebrauch [1, 3]. Von Leonowas wahrscheinlich wichtigste Station war das Züricher „Labörli“, das von Monakow 1885 in seinen privaten Räumen eingerichtet hatte. Dort arbeitete sie seit 1892 tagsüber unbeaufsichtigt und die Ergebnisse wurden nur abends kontrolliert ([7]; Tab. 1; vL 1893b). Sie leistete „recht wertvolle technische Arbeit (Schnittserien durch pathologische Objekte und Präparate)“, die von Monakows eigenen Werken nach seiner Aussage zugutekamen [7]. Zitiert wurden von Leonowas frühe Arbeiten in den Folgejahren von zahlreichen wichtigen deutschsprachigen und internationalen Autoren, darunter Arnold Pick [10]. Auch Freud besaß zeitweise eine ihrer Publikationen [2].

**Table 1:** 

1890	Ein Fall von Anencephalie. Ueber den feineren Bau des Rückenmarkes eines Anencephalus. Archiv fuer Anatomie und Physiologie (Anat. Abth.) 403–422
1893a	Ueber das Verhalten der Neuroblasten des Occipitallappens bei Anophthalmie und Bulbusatrophie und seine Beziehungen zum Sehact. Archiv fuer Anatomie und Physiologie (Anat. Abth.) 308–318
1893b	Zur pathologischen Entwickelung des Centralnervensystems. (ein Fall von Anencephalie combiniert mit totaler Amyelie). Neurologisches Centralblatt 7: 218–227; 8: 263–267
1894a	Contribution a l’etude de l’evolution pathologique du systeme nerveux. Anencephalie totale combine avec une amyelia et un rhachischisis totaux chez un embryon humain. Moscou Soc Nat Bull 7: 191–198
1894b	Die Sinnesorgane und Ganglien bei Anencephalie und Amyelie. (zweiter Fall von totaler Amyelie). Verhandlungen der Gesellschaft deutscher Naturforscher und Aerzte, 2. Hälfte, Neurologisches Centralblatt 13: 176–177
1896	Beiträge zur Kenntniss der secundären Veränderungen der primären optischen Centren und Bahnen in Fällen von congenitaler Anophthalmie und Bulbusatrophie bei neugeborenen Kindern. Archiv für Psychiatrie und Nervenkrankheiten 28: 53–96
1897	Einige Bemerkungen zu im Archiv für Psychiatrie Bd. 28, H. 1, erschienenen Abhandlung: Beiträge zur Kenntnis der secundären Veränderungen der primären optischen Centren und Bahnen in Fällen von congenitaler Anophthalmie und Bulbusatrophie bei neugeborenen Kindern. Moscou Soc Nat Bull 10: 570–574
1904	Zur pathologischen Entwickelung des Centralnervensystems (neue Beiträge). Ein Fall von Cyclopie combinirt mit Mikro- und Arhinencephalie. Archiv für Psychiatrie und Nervenkrankheiten 38: 862–894
1905	Missbildungen und Entwicklungsstörungen im Gehirn (Abstract). Jahresbericht über die Leistungen und Fortschritte auf dem Gebiet der Neurologie und Psychiatrie
1907	Zur pathologischen Entwickelung des Centralnervensystems. Das Verhalten der Rinde der Sulci calcarina in einem Falle von Mikrophthalmia und Amelie (Amputation spontanee). Monatsschrift für Psychiatrie und Neurologie 21:176
1908	Zur pathologischen Entwickelung des Centralnervensystems. Ein Fall von Amelia (Amputatio spontanea) (neue Beiträge). Archiv für Psychiatrie und Nervenkrankheiten 43: 1218–1252
1909	Zur pathologischen Entwickelung des Centralnervensystems. Das Verhalten der Rinde des Sulci calcarina in einem Falle von Mikrophthalmia bilateralis congenita. Archiv für Psychiatrie und Nervenkrankheiten 45: 77–91

Ihrer ersten Studie über Anenzephalie aus dem Jahr 1890 waren weitere zu Anophthalmie und Bulbusatrophie, Anenzephalie mit Amyelie, zwei zu Anenzephalie und Rachischisis, zu Anophthalmie u. a. gefolgt (Tab. 1). Wenngleich mit einer zeitlichen Lücke von 1897 bis 1904, nach der sie als Olga von Leonowa-von Lange firmierte, war sie also dem Thema der Neuroembryologie zwanzig Jahre treu geblieben. Sie wurde dabei nicht müde auf die besonders mühsame Arbeit hinzuweisen. Von Monakow hatte ihr zunächst in Zürich Präparate zur Verfügung gestellt (vL 1893b, 1896), sie bedankte sich aber auch bei Professor Fenomenoff, seit 1899 Direktor der Universitätsklinik für Gynäkologie und Geburtshilfe in Sankt Petersburg (vL 1908), und bei einem „Collegen von mir“ (vL 1909).

## Fußnoten und Seitenhiebe

Zwölf Jahre nach dem großen ersten Auftritt meldete sich Olga von Leonowa auf der Wanderversammlung des Vereins für Psychiatrie und Neurologie im Oktober 1906 in Wien zurück [11]. Sie sprach am Vormittag des 6. Oktober „Ueber das Verhalten der Rinde der Calcarina in einem Falle von Mikrophthalmia und Amelie (Amputation spontanee)“. Im Gegensatz zu den anderen Beiträgen finden sich hier weder eine Zusammenfassung, noch Hinweise auf eine Diskussion [11]. Ein Grund hierfür mag gewesen sein, dass ihr Beitrag nicht angemeldet war und der Vorsitzende Heinrich Obersteiner sie dennoch ins Programm aufnahm (vL 1909). In ihrer dazugehörigen und insgesamt letzten Publikation (vL 1909) gibt sie als Affiliation München an. In den Annalen der Münchner Universität ist von Leonowa nicht verzeichnet (die weniger produktive Emma Mooers schon [3]). Franz Nissl hatte sie 1904 von Heidelberg aus Emil Kraepelin in München wenig charmant angekündigt [9]: „Frl. Leonowa ist eingetroffen. Ein mutziges – so sagen wir in Bayern^1^ – Frauenzimmer, rein menschlich gesprochen. Eine Art Zwischenstufe zwischen Mann und Weib. Ich glaube, dass sie guten Willen hat. Aber maximal eng begrenzter geistiger Horizont. Ausser der Rinde der fissura calcarina scheint sie überhaupt Nichts in der Welt zu interessieren …“.

Einige ihrer Fußnoten belegen von Leonowas gespanntes Verhältnis zur deutschen Sprache und ihre geringe Neigung sich Freunde im Kollegenkreis zu machen. „… Die Verhältnisse sind aber weit complicirter, wie sich Herr Zingerle vorstellt. Wenn man aber schon so geneigt ist die Zahl der Fälle dem Inhalte gegenüber vorzuziehen, wie es Herr Zingerle auch thut, so möchte ich ihm mit Goethe den Rath geben ‚Braut ein Ragout von andrer Schmaus‘“ (vL 1904; S. 892). Damit gemeint ist wohl, dass Zingerle sich an ihren Erkenntnissen bereicherte, ohne sie verstanden zu haben. Daraufhin setzte sich Hermann Zingerle kurz, heftig und mit überzeugenden Argumenten zur Wehr [13]: „… Die ungenaue Auffassung, gepaart mit Oberflächlichkeit des Urtheils und Mangel der Kritik finden aber noch eine interessante Ergänzung in dem etwas ungewöhnlichen Tone, der gegen mich gerichteten Polemik, auf den einzugehen mich sowohl der gute Geschmack, als auch die Achtung der traditionellen Stellung, die dieses Archiv in unserer Fachliteratur einnimmt, hindert.“ Hierbei ist allerdings zu bedenken, dass in der damaligen Zeit Diskussionen in Fachzeitschriften gelegentlich durchaus polemisch und persönlich geführt wurden.

Sie verstand es aber auch, nicht nur einzelne Kollegen, sondern die ganze Zunft der Psychiater gegen sich aufzubringen (vL 1909, S. 88–89; kursiv und Fettdruck nach dem Original): „… Die Psychiatrie hat keinen Anspruch auf die Enträhselung (!) jenes Gesetzes, sie ist nicht im Stande, das Functionsprincip der corticalen Nervenzelle zu entdecken und sie wird ihn auch nie beherrschen, denn man kann den Geist doch nicht im Gebiete aufsuchen, wo er fehlt. Man soll ihn da suchen, wo er sich ausspricht: in den socialen Wissenschaften, im allgemeinen Recht der Völker. … *meine vieljährige Arbeit hat mich zu dem Entschluss gebracht, dass* **jene Gleichförmigkeit, welche man im Rechtsbewusstsein des Volkes bei allen Völkern der Welt in ganz ähnlicher, gleicher Weise begegnet, eben das Functionsprincip der corticalen Nervenzelle bildet.** …“. Die Argumentation wird über zwei Seiten unter emphatischer Zuhilfenahme von Plato und Sokrates, Wilhelm Griesinger, Moritz Benedikt und Julius Wagner-von Jauregg fortgesetzt, ohne größere inhaltliche Klarheit zu stiften, und schließt mit einem heftigen und ausführlichen Seitenhieb auf Salomon Henschen. Der Verdacht, dass ihre hochfliegenden Gedanken auch vom Geist der Revolution beflügelt waren, ist nicht auszuschließen.

## Der Verdacht

„Russin Olga Leonowa heute festnehmen und ins Amtsgefängnis. Haussuchung nach Vorschrift vornehmen“ – Telegramm aus Säckingen an die Gendarmeriestation Kleinlaufenburg vom 4. August 1914, 5:53 [5, S. 3]. Olga von Leonowa wurde vom 4. August bis Ende November 1914 in Säckingen in Schutz- und Sicherungshaft genommen. 1910 hatte sie mit 35.000 Goldmark eine Villa im südbadischen Grenzort Kleinlaufenburg nahe bei einem Eisenbahntunnel erworben und sich dadurch am Beginn des 1. Weltkriegs als mögliche russische Agentin verdächtig gemacht [5, S. 16]. Im Verhör am 25. November 1914 wurde unter anderem notiert, sie sei am 21. Januar 1851 in Moskau geboren und in den letzten Jahren als Assistentin in Heidelberg, München und zuletzt im Jahr 1909 in Freiburg bei der psychiatrischen Klinik tätig gewesen. Dies habe sie gesundheitshalber aufgegeben und sich aus ihrem Privatvermögen einen Wohnsitz gekauft, um zurückgezogen leben zu können [5, S. 21 f.]. Am gleichen Tag wurde an das Grossherzogliche Ministerium des Inneren gemeldet, sie habe mit niemand im Ort Verkehr und führe ein absonderliches Dasein, „auch reduziertes Aussehen, kurz geschnittene Haare, wenig sorgfältige alte Kleidung etc machten sie zu einer eigentümlichen Erscheinung. … Ihr Gesundheitszustand war nach Angaben des Gr. Bezirksarztes immer ein guter, nur soll sie stark hysterisch veranlagt sein“ [5, S. 27 f.]. Am 26. Januar 1915 stellte der praktische Arzt H. Möschler folgendes Attest aus [5, S. 47 f.]: „… am 14. Januar 1913 erlitt Frl. Dr. von Leonowa eine Apoplexia cerebralis (Hirnschlag), deren Folgen sich lange Zeit bemerkbar machten … wegen dieser Erkrankung brachte sie einige Zeit in meinem Nervensanatorium in Heidelberg zu … Hände und Vorderarme chronisches Ekzem … Weinkrämpfe, Zittern der Hände, Pulszahl >100/min. Wie nun die Patientin angibt, dass sie als Folge der Belästigungen von Fabrikarbeitern und Schulkindern immer in hochgradigen Aufregung versetzt werde, sobald sie sich öffentlich zeigen müsse.“ Es wird daraufhin entschieden, dass die tägliche Meldung bei der Gendarmeriestation zu Zeiten erfolgen darf, zu denen eine Begegnung mit Schulkindern und Fabrikarbeitern weniger wahrscheinlich sei. Am 12. April 1916 wurde die Übersiedlung von Frau von Leonowa in die Schweiz genehmigt. Sie reiste am 29. April 1916 ab, ohne weitere Spuren zu hinterlassen.

## Katamnese

Olga von Leonowa war – vermutlich mit Hilfe ihres Förderers – die Gelegenheit zu einem großartigen Start geboten worden. Von Monakow saß am gleichen Tag danach etwas steif zwischen den Kollegen Kahlbaum und Forel (Abb. 1). Er war der einzige, der seiner Schülerin in der Diskussion sekundiert hatte. Von Leonowa blieb dem Thema schwerer zerebraler Missbildungen während ihrer ganzen Karriere verhaftet und war von der grundsätzlichen Bedeutung ihrer Erkenntnisse auch für andere Wissenschaftsbereiche bis zu ihrer letzten Publikation überzeugt. Vergleicht man die methodische Herangehensweise (Fallzahlen, Aufarbeitung, Darstellung), so ist bei ihr im Gegensatz zu vielen Zeitgenossen wenig Weiterentwicklung festzustellen. In ihren Veröffentlichungen betonte sie immer wieder die besondere Mühe, die sie auf die Arbeiten verwendet hatte, scheint misstrauisch über ihr geistiges Eigentum zu wachen, zeigt aber auch eindrucksvolles Selbstbewusstsein und keine Scheu vor öffentlicher Auseinandersetzung. Von Leonowa war nicht das einzige Opfer von Franz Nissls spitzer Zunge und Feder, aber Nissl war auch nicht der einzige, der Anlass hatte seinen Unmut zu bekunden. Einiger Schaden wäre wohl durch die schützende Hand von Herausgebern vermieden worden. Die schriftlichen Zeugnisse von und über Olga von Leonowa liefern nicht nur Hinweise auf Phasen einer epochentypischen Reiselust und ihre Hochstimmung [1, 4], sondern auch auf Perioden anhaltender Niedergeschlagenheit und Einsamkeit. Am Ende ihrer Karriere schien sie – vielleicht mit Ausnahme ihres Arztes – keine Verbündeten mehr zu haben. Dass sich Wladimir I. Uljanow, genannt Lenin, wenige Tage vor ihrem Verschwinden im 50 km entfernten Zürich eine Wohnung genommen hatte, ist wahrscheinlich ein Zufall.

## Footnotes

1 Nicht überall. Etymologisch am ehesten „motzig, verdrießlich“.

## References

[1] Creese MRS (2015). Ladies in the laboratory IV: Imperial Russia’s women in Science, 1800–1900: a survey of their contributions to research.

[2] Davies JK, Fichtner G (2004). Freud’s Library; a comprehensive catalogue (Item 2162).

[3] Förstl H (2020). Emma Wilson Mooers (1858–1911): die Neuropathologin an Aloys Alzheimers Seite. Nervenarzt.

[4] Hacking I (1998). Mad Travellers: reflections on the reality of transient illnesses.

[5] Landesarchiv Baden-Württemberg, Staatsarchiv Freiburg (1914–16) Behandlung von Angehörigen feindlicher Staaten in Kleinlaufenburg, insbesondere der Russin Dr. Olga von Leonowa. Findbuch B733/1, Nr. 6984, Landratsamt Säckingen.

[6] Molnar M (2011). Mysteries of nature. Psychoanalysis & History.

[7] v Monakow K (1970). Vita mea. Mein Leben.

[8] Neue Freie Presse (1894) Deutscher Naturforschertag. No. 10809, S. 6, Wien

[9] Nissl F, Burgmair W, Engstrom EJ, Weber MW (1904). Brief an Emil Kraepelin. Kraepelin in München I, 1903–1014.

[10] Pick A (1898). Beiträge zur Pathologie und pathologischen Anatomie des Centralnervensystems mit Bemerkungen zur normalen Anatomie desselben.

[11] Pilcz A (1906). Wanderversammlung des Vereins für Psychiatrie und Neurologie in Wien. Monatsschr Psychiatr Neurol.

[12] Redlich E (1894). Bericht über die 66. Versammlung deutscher Naturforscher und Aerzte in Wien vom 24. Bis 30. September 1894. Neurol Centralbl.

[13] Zingerle H (1905). Erwiderung auf den Aufsatz von Dr. O. von Leonowa-v. Lange: Zur pathologischen Entwickelung des Centralnervensystems. Arch Psychiatr Nervenkr.

